# Unintended Electrical Isolation of the Left Atrial Appendage due to Anatomical Misidentification During Pulsed‐Field Ablation: A Case Report

**DOI:** 10.1002/joa3.70188

**Published:** 2025-10-22

**Authors:** Naoki Matsumoto, Kenji Shimeno, Masanori Matsuo, Yukio Abe, Daiju Fukuda

**Affiliations:** ^1^ Department of Cardiology Osaka City General Hospital Osaka Japan; ^2^ Department of Cardiovascular Medicine Osaka Metropolitan University Graduate School of Medicine Osaka Japan

**Keywords:** atrial fibrillation, electrical isolation, left atrial appendage, pulmonary vein isolation, pulsed field ablation

## Abstract

During pulsed‐field ablation, unintended left atrial appendage isolation occurred due to anatomical misidentification with the left superior pulmonary vein. Left atrial appendage potentials improved after 1 month. This case highlights procedural tips to avoid this complication and emphasizes post‐procedural monitoring and management strategies.

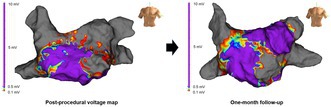

## 1 Case Presentation

**FIGURE 1:**
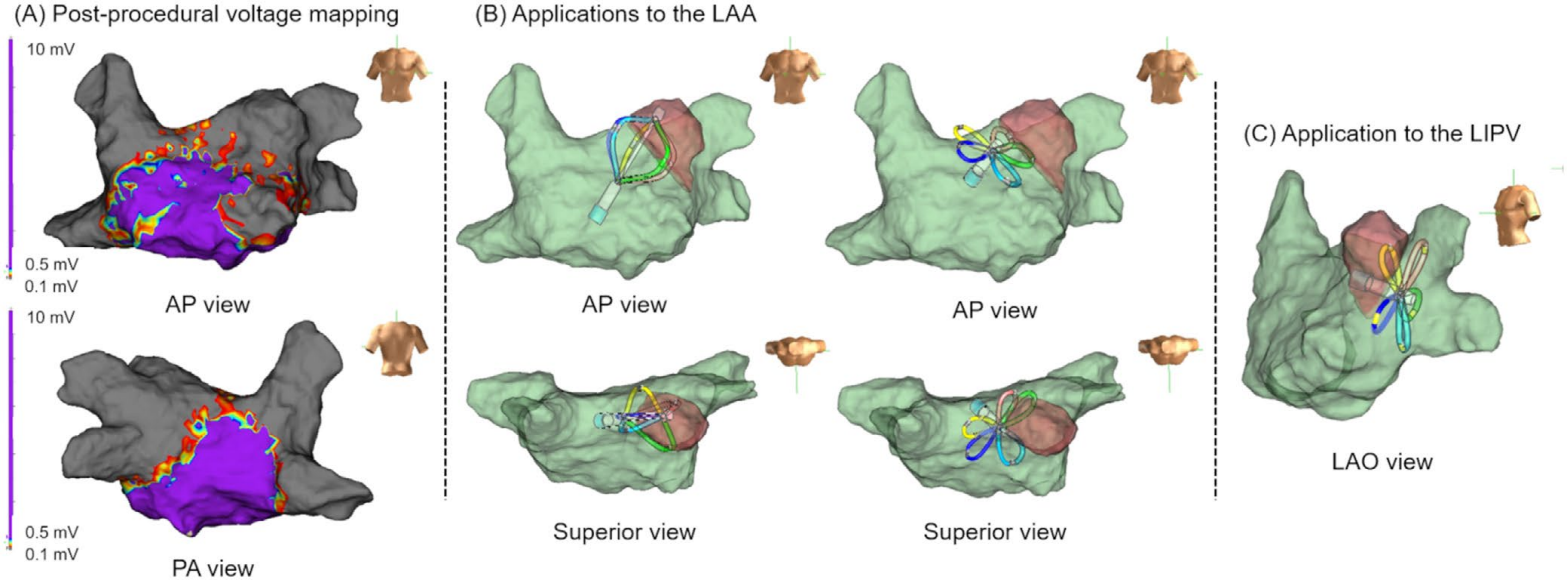
(A) Post‐procedural left atrial voltage mapping. The voltage map shows unintended isolation of the left atrial appendage (LAA) and inadvertent completion of the roof line. (B) The images show the sites where applications were delivered to the LAA using the basket and flower configurations. A retrospective analysis using the EnSite NavX system (Abbott Laboratories) revealed that the flower configuration applications to the LAA had been delivered more proximally than the left pulmonary veins. The basket configuration applications appeared to achieve better contact from the ridge to the roof of the left superior pulmonary vein. (C) The image shows the site where application was delivered to the left inferior pulmonary vein (LIPV) using the flower configuration. The flower configuration applications to the LIPV covered a wide area of the left pulmonary veins. AP, anterior–posterior; LAA, left atrial appendage; LAO, left anterior oblique; LIPV, left inferior pulmonary vein; PA, posterior–anterior.

A 24‐year‐old man underwent pulsed field ablation (PFA) for symptomatic, recurrent paroxysmal atrial fibrillation (AF). Pre‐procedural transthoracic echocardiography revealed a left atrial diameter of 27 mm. Contrast‐enhanced computed tomography demonstrated anteroposterior compression of the left atrium and close proximity between the left superior pulmonary vein (LSPV) and left atrial appendage (LAA). The FARAPULSE system (Boston Scientific, Natick, MA, USA) was used for ablation, and EnSite NavX (Abbott Laboratories, Chicago, IL, USA) served as the 3D mapping system. Mapping was performed with the Advisor Circular Mapping Catheter SE (Abbott Laboratories). Per protocol, four applications in basket configuration were delivered at the ostium and four in flower configuration at the antrum for each pulmonary vein (PV). Post‐procedural voltage mapping revealed a completed roof line with low‐voltage areas in the anterior and lateral walls. Notably, electrical signals in the LAA were absent (Figure 1A, Video S1). While the initial absence of signals in the LAA raised suspicion of poor catheter contact, repeat mapping by two independent operators confirmed true electrical isolation. Fluoroscopic review revealed that the LSPV and LAA had been misidentified, resulting in unintended LAA isolation.

Due to the risk of thrombus formation within the electrically isolated LAA, anticoagulation was switched from edoxaban to warfarin with a target PT‐INR of 2.5 to 3.0. Given the long‐term bleeding risk and embolic potential, LAA closure or surgical resection was considered. However, since the patient's HAS‐BLED score was 0, percutaneous LAA occlusion was not indicated under current domestic guidelines. Surgical resection was therefore initially contemplated.

**FIGURE 2:**
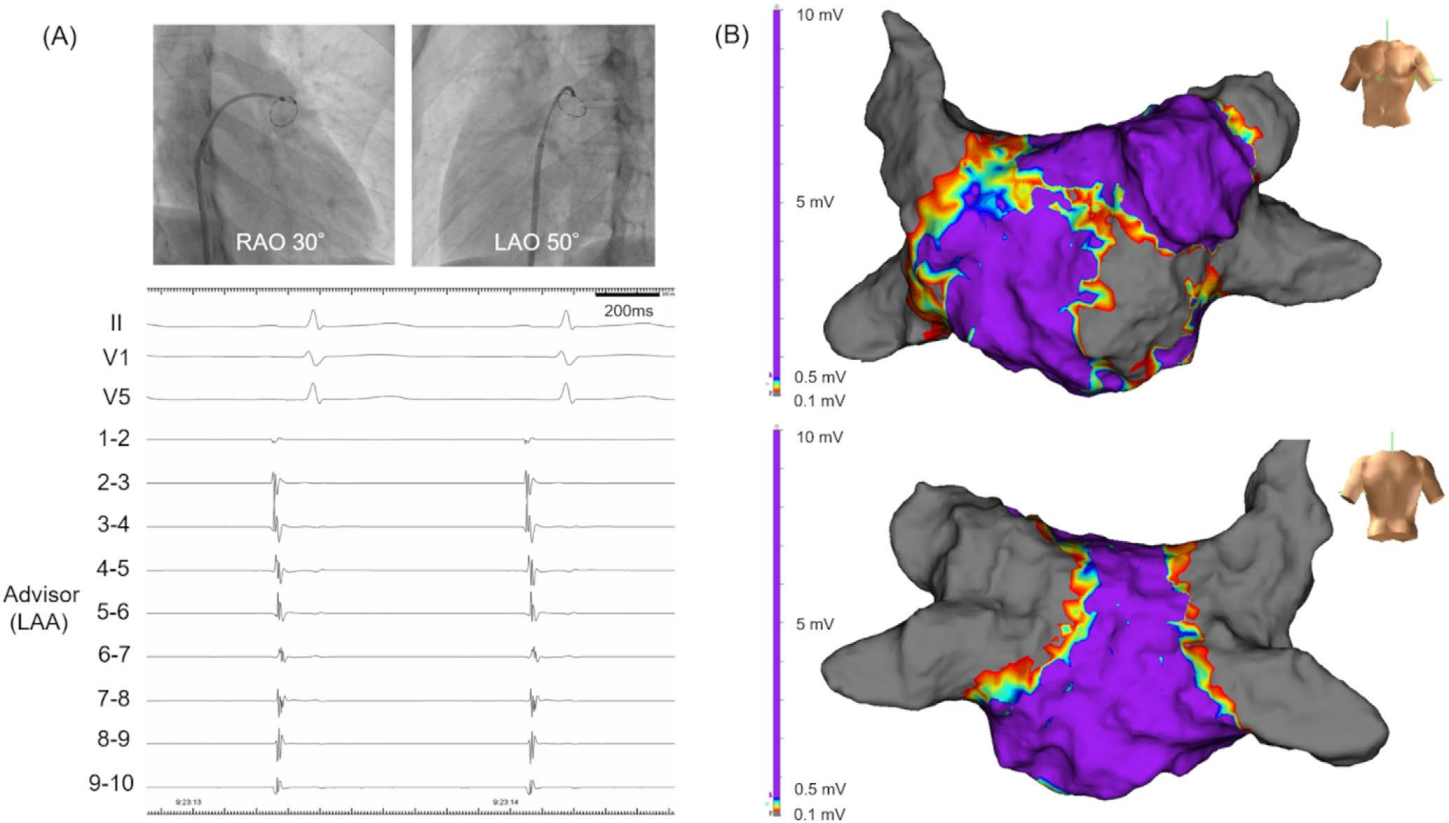
Fluoroscopic images and voltage mapping 1 month after inadvertent isolation of the left atrial appendage (LAA). (A) A 10‐polar circular mapping catheter shows reappearance of LAA potentials. (B) Voltage mapping demonstrates resolution of LAA isolation and improvement of voltage in the roof area. RAO, right anterior oblique; Abbreviations are as above.

To assess thrombus formation, LAA function, and potential improvement of conduction, follow‐up transesophageal echocardiography (TEE) and electrophysiological study (EPS) were performed 1 month later. TEE showed no thrombus, with LAA inflow velocity at 69 cm/s and biphasic flow (Figure S1). Voltage mapping revealed a small low‐voltage area in the central anterior wall, and although conduction delay in this region was suggested, signal reappearance was observed in the roof and LAA (Figure 2, Video S2). No PV reconnection was observed, including in the LSPV. LAA contraction was confirmed on contrast imaging (Video S3). Compared with the pre‐procedural electrocardiogram, the one‐month post‐procedural electrocardiogram showed decreased P‐wave amplitude in the inferior leads and the appearance of negative components in lead V1, while P‐wave duration remained unchanged (Figure S2). These findings suggested that, although the overall atrial activation time was preserved, residual impairment of the anterior and lateral walls may have affected the overall left atrial activation pattern. Given the improvement of LAA conduction and function, surgical resection was deemed unnecessary. The patient was transitioned back to edoxaban 60 mg once daily. At six‐month follow‐up, there were no recurrences of AF or thromboembolic events. As his CHADS_2_ score remains 0, discontinuation of anticoagulation is under consideration with close monitoring.

## 2 Discussion

LAA isolation is generally not recommended in standard AF ablation procedures due to the associated risk of thromboembolism [1, 2]. Unintended LAA isolation using the FARAPULSE system has been previously reported in a patient with a narrow anteroposterior left atrial diameter [3]. In contrast to the previously reported case, the LAA isolation in the present case represents a complication caused by misidentification of the LAA location; however, to our knowledge, this is the first report to provide detailed post‐procedural follow‐up. Given that PFA procedures are often performed under fluoroscopic guidance, this complication may occur in the future. Therefore, reporting this case has clinical significance, not only in raising awareness of this potential complication but also in illustrating appropriate strategies for subsequent follow‐up.

**FIGURE 3:**
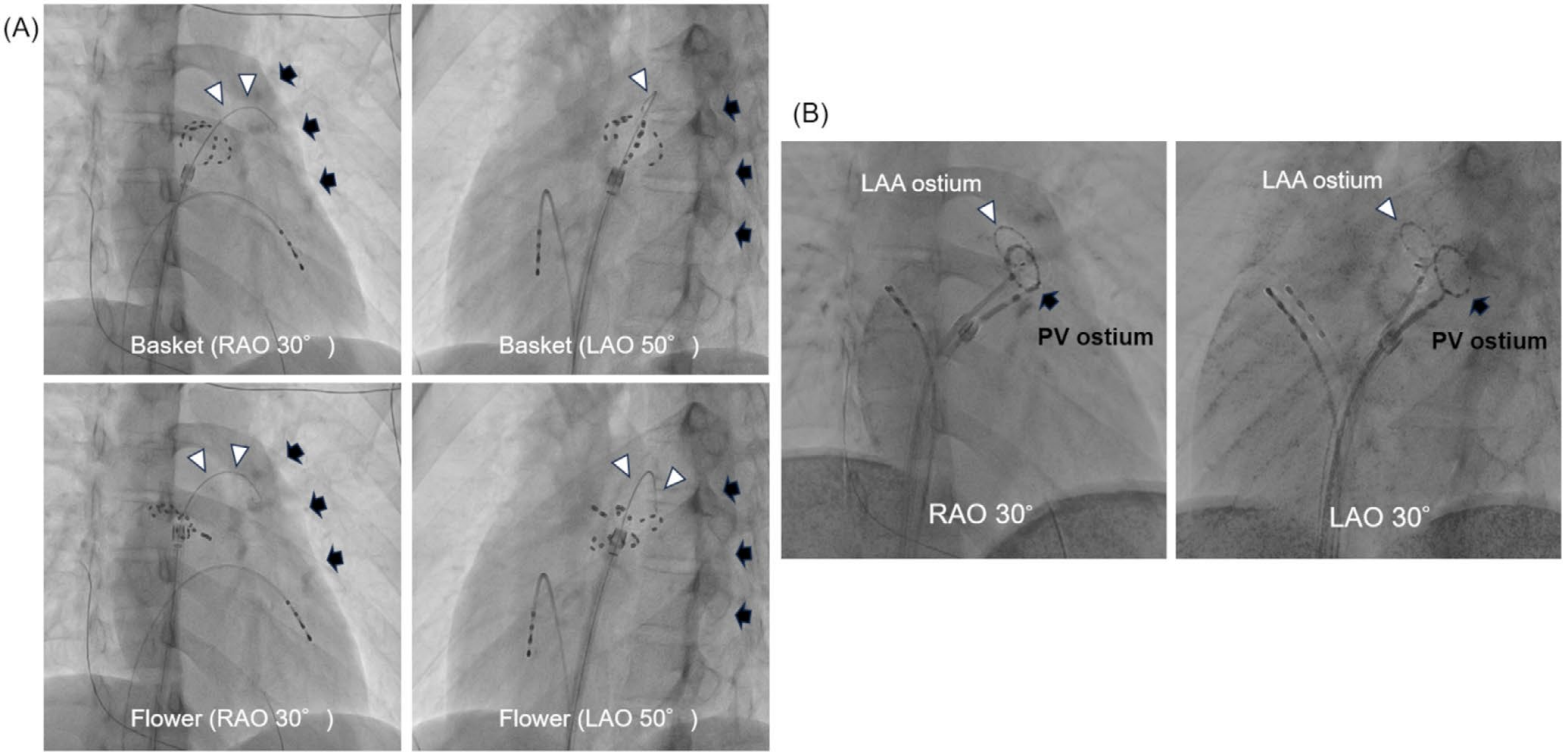
(A) Fluoroscopic image showing the relationship between the guidewire and the cardiac silhouette during attempted ablation of the left superior pulmonary vein (LSPV). During applications with both the basket‐ and flower‐type configurations, the guidewire appeared to remain within the cardiac silhouette without extending into a pulmonary vein branch. The arrowhead indicates the wire's course, and the black arrow indicates the cardiac silhouette. (B) Overlay image showing the positional relationship between the ring catheters placed at the ostia of the left atrial appendage (LAA) and LSPV. The LAA and LSPV are located in close proximity. PV, pulmonary vein; Abbreviations are as above.

In the present case, ablation energy was mistakenly delivered to the ostium of the LAA, which had been misidentified as the LSPV. Unlike a previously reported case [3], the guidewire was not advanced beyond the cardiac silhouette into a pulmonary vein branch—this was the most critical procedural error. This procedural error stemmed from the misidentification of the LAA as the LSPV, underscoring the difficulty in fluoroscopic discrimination without contrast injection or intracardiac echocardiography (ICE). Contributing factors included the patient's narrow anteroposterior atrial dimension, as well as the close anatomical proximity and similar angulation of the LSPV and LAA (Figure 3A,B). To prevent misidentification of the LAA, it is essential to confirm guidewire placement within a pulmonary vein branch using multiple fluoroscopic views. If this proves difficult, or in patients with a small left atrial volume, as in the present case, left atrial angiography or ICE should be used not only to confirm guidewire position but also to verify catheter contact location.

Immediately after ablation, the electrical signals in the roof and LAA were abolished but reappeared 1 month later. Adequate catheter contact during PFA is associated with deep lesions, whereas insufficient contact may result in superficial lesions despite initial electrogram reduction [4]. Lesion regression over time following PFA has also been reported [5]. Our procedural emphasis on posterior wall contact likely led to suboptimal contact with the anterior wall and LAA, where the reappearance of electrograms was observed. With PFA, catheter instability due to phrenic nerve stimulation or coughing can also impair contact, contributing to incomplete lesions and the partial improvement of electrical signals, as observed in this case. These findings suggest that early post‐procedural isthmus‐like conduction block may regress over time, and additional ablation may not be necessary if the risk of atrial tachycardia is low. In contrast, electrical isolation of the LSPV, to which no intentional applications had been delivered, was maintained at the one‐month follow‐up. A retrospective analysis using the EnSite NavX revealed that the flower configuration applications to the left inferior pulmonary vein (LIPV) covered a wide area of the left pulmonary veins (Figure 1B,C). Although the flower configuration made contact with a more proximal portion of the LAA, the basket configuration appeared to achieve better contact from the ridge to the roof of the LSPV. These findings suggest that the combination of flower configuration applications to the LIPV and basket configuration applications involving the LAA may have contributed to the durable isolation of the LSPV.

In the present case, resumption of conduction to the LAA was observed; however, if LAA isolation persists, subsequent treatment strategies should be carefully considered. Even in patients who maintain sinus rhythm and receive appropriate anticoagulation therapy, thrombus formation in the LAA has been reported following isolation [1, 2]. In addition, a previous study reported that percutaneous left atrial appendage closure resulted in fewer stroke events and less LAA thrombus formation compared to continued oral anticoagulation in patients with LAA isolation [6]. Therefore, appropriate strategies—such as oral anticoagulation, LAA closure, or surgical excision—should be considered on a case‐by‐case basis.

In cases of unintended LAA isolation, clinicians should consider the possibility of spontaneous improvement in LAA conduction and function. This may obviate the need for surgical or percutaneous interventions. Evaluation of LAA flow velocity and pattern via TEE, in combination with EPS and angiographic findings, can assist in clinical decision‐making. However, our patient was young and likely had preserved atrial substrate; it remains uncertain whether similar outcomes can be expected in older patients or those with advanced atrial remodeling.

## 3 Conclusions

Given that PFA is frequently guided by fluoroscopy alone, clinicians should remain vigilant for inadvertent LAA isolation and ensure appropriate diagnostic and management strategies are in place when such complications arise.

## Consent

Informed consent was obtained from the patient to publish the case report.

## Conflicts of Interest

The authors declare no conflicts of interest.

## Supporting information


**Figure S1:** Transesophageal echocardiography demonstrated the left atrial appendage (LAA). No thrombus was observed within the LAA, and the LAA flow velocity was normal at 69 cm/s, with a normal biphasic flow pattern. LAA, left atrial appendage.

**Figure S2:** Pre‐procedural and one‐month follow‐up electrocardiograms. At follow‐up, the P‐wave amplitude was slightly reduced in the inferior leads and negative components appeared in lead V1; however, the P‐wave duration remained unchanged compared with the pre‐procedural recordings.

**Video S1:** Voltage map with sparkle propagation immediately after the ablation procedure.

**Video S2:** Voltage map with sparkle propagation at the 1‐month follow‐up.

**Video S3:** Left atrial appendage angiography at the 1‐month follow‐up.

## Data Availability

The data underlying the results are available in this article.
