# Supplementary figures and images for: Unintended Electrical Isolation of the Left Atrial Appendage due to Anatomical Misidentification During Pulsed‐Field Ablation: A Case Report
Source: J Arrhythm. 2025 Oct 22;41(5):e70188. doi: 10.1002/joa3.70188 (PMC12541545; doi:10.1002/joa3.70188)

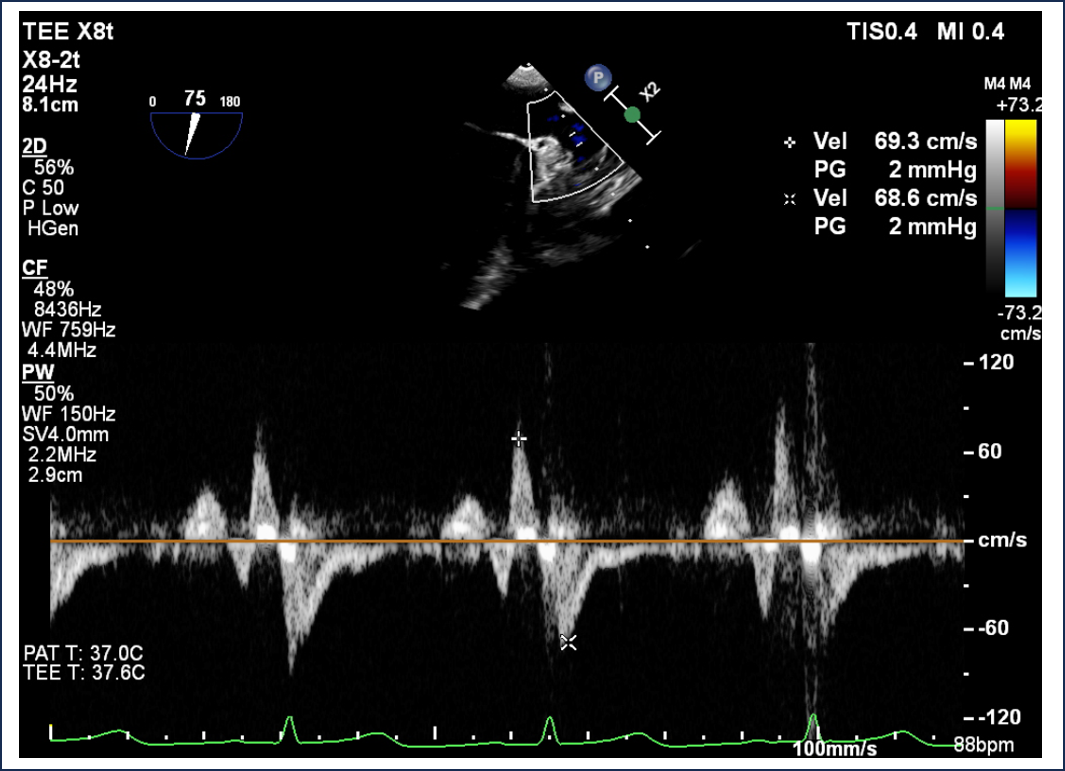

Supplement: Supplementary file 1 — Figure S1: Transesophageal echocardiography demonstrated the left atrial appendage (LAA). No thrombus was observed within the LAA, and the LAA flow velocity was normal at 69 cm/s, with a normal biphasic flow pattern. LAA, left atrial appendage. [file JOA3-41-e70188-s005.tif]

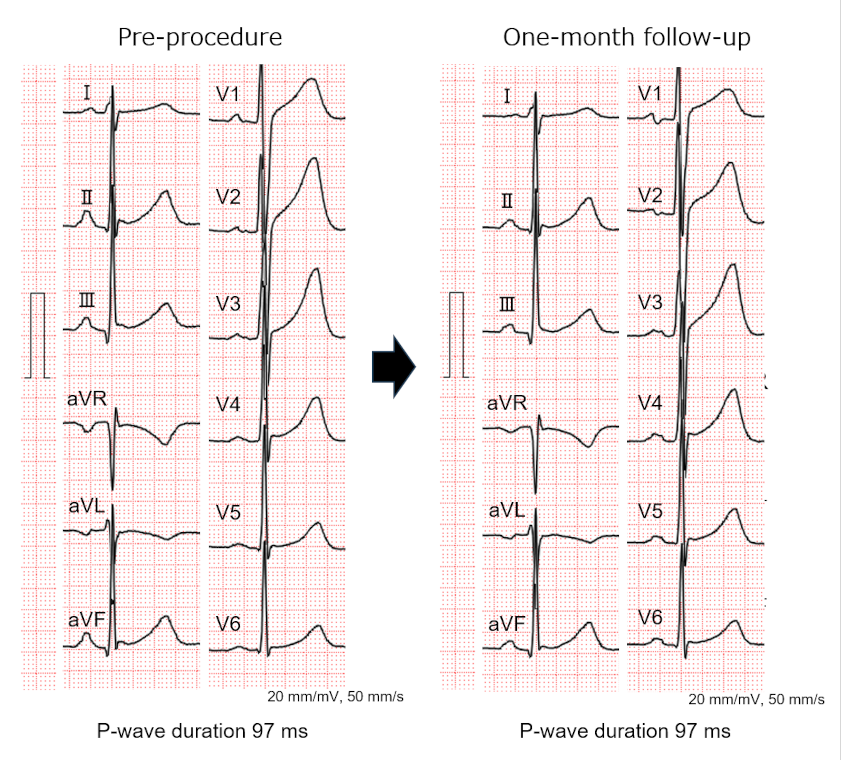

Supplement: Supplementary file 2 — Figure S2: Pre‐procedural and one‐month follow‐up electrocardiograms. At follow‐up, the P‐wave amplitude was slightly reduced in the inferior leads and negative components appeared in lead V1; however, the P‐wave duration remained unchanged compared with the pre‐procedural recordings. [file JOA3-41-e70188-s001.tif]
